# Prevalence of Antibiotic Resistance in Older Adults and Alzheimer’s Disease Patients: A Systematic Review and Meta-Analysis

**DOI:** 10.3233/ADR-240057

**Published:** 2024-09-10

**Authors:** Namra Vinay Gohil, Fabio Fuentes Gandara, Harshal Gohil, Swathi Gurajala, David Chinaecherem Innocent, Tadele Tesfaye, Domenico Praticò

**Affiliations:** a Medical College Baroda and SSG Hospital Vadodara, Gujarat, India; b Department of Natural and Exact Sciences, Universidad de la Costa, Barranquilla, Colombia; c Department of Community Medicine, GMERS Medical College, Panchmahal, Godhra, Gujarat, India; d College of Applied Medical Sciences in Jubail, Imam Abdulrahman Bin Faisal University, Dammam, Saudi Arabia; e Department of Public Health, Federal University of Technology, Owerri, Imo, Nigeria; f CareHealth Medical Practice, Addis Ababa, Ethiopia; g Alzheimer’s Center at Temple, Lewis Katz School of Medicine, Temple University, Philadelphia, PA, USA

**Keywords:** Alzheimer’s disease, antibiotic resistance, meta-analysis, older adults, prevalence, systematic review

## Abstract

**Background:**

Antibiotic resistance is a global health concern, and its prevalence among older adults and Alzheimer’s disease (AD) patients is gaining attention. Understanding the extent of antibiotic resistance in these populations is critical for designing targeted interventions.

**Objective:**

The objective of this systematic review and meta-analysis was to determine the prevalence of antibiotic resistance in older adults and AD patients with a focus on quantitative studies in order to provide comprehensive insights into the current landscape.

**Methods:**

To identify relevant studies, we conducted a thorough search of the PubMed, Scopus, CINAHL, and Web of Science databases. Only studies involving adults and AD patients, published in English, and reporting quantitative data on antibiotic resistance prevalence were considered. The Risk of Bias In Non-randomized Studies of Interventions (ROBINS-I) tool was used to assess quality. The data was summarized by using Revman 5.4.1.

**Results:**

A total of six studies met the final criteria for selection and results from the meta-analysis found a pooled prevalence odds ratio of OR = 1.27 (95% CI: [0.99, 1.63], *Z* = 1.87, *p* = 0.06). The studies showed significant heterogeneity (I2 = 100%, *p* < 0.00001), emphasizing the need for cautious interpretation.

**Conclusions:**

The findings indicate a potential trend of increased antibiotic resistance in older adults and AD patients, though statistical significance was not achieved for both. The significant heterogeneity highlights the complexity of resistance patterns in these populations, necessitating additional research for tailored interventions.

## INTRODUCTION

Antibiotics have transformed modern medicine in the treatment of bacterial infections;^1^ however, the emergence and spread of antibiotic resistance (AR) has become a global health concern,^2,3^ highlighting the critical need to address it.^4^ The ability for bacteria to resist the effects of an antibiotic to which they were previously susceptible^5–7^ or evolve and develop the mechanisms to withstand exposure to drugs (antibiotics) designed to kill them, renders medication efforts ineffective.^8,9^ According to estimates, AR was responsible for nearly 5 million deaths in 2019 worldwide.^10^ If appropriate measures are not implemented, it is anticipated that this number will increase to between 10 and 44 million by the year 2050.^1,6,11^ Over 2.8 million cases of antibiotic-resistant infections happen each year in the United States, leading to more than 35,000 deaths.^12^ The annual cost of resistant infections is estimated to be $20 billion in excess health-care costs in the United States,^7^ accounting for over 30% of antimicrobials hospital pharmacy budgets. In the European Union, the cost is over 1.6 billion euros.^10^

Risk factors which contribute to the development and spread of AR, include but not limited to inappropriate use of antibiotics, inadequate infection prevention and control, suboptimal sanitary conditions,^7,11,12^ and conditions that necessitate frequent use of antibiotics, such as diabetes.^13–15^ It is therefore crucial to investigate the implications for vulnerable populations with coexisting conditions, particularly those with neurodegenerative disorders with dementia such as Alzheimer’s disease (AD).^13–19^

Millions of AD patients worldwide are vulnerable to a variety of health issues,^20,21^ and the combination of AR increases their vulnerability.^22^ Complications such as increased infection severity, longer hospitalizations, higher healthcare costs, and compromised treatment outcomes are significant challenges for AD patients with AR.^23^ The challenges extend beyond just medical issues, encompassing significant socioeconomic burdens.^4^ Medical complexities refer to the intricate interplay of managing both AR and AD, which can complicate treatment plans and patient care. Socioeconomic burdens include the increased healthcare costs, the need for specialized care, and the impact on the quality of life for both patients and caregivers. Addressing these factors is essential to improve patient outcomes and provide effective, comprehensive care.^4^

Existing studies report varying levels of AR in AD patients, with distinct incidence rates across regions: 15% in Africa, 19.38% in Europe.^17,21,24–27^ However, no comprehensive review exists on AR prevalence in AD patients, especially those with co-morbid diabetes, a known AR risk factor due to its impact on immune responses.^22^ Region-specific measures are crucial: Africa could focus on improving antibiotic stewardship and infection control, given its lower rate, while Europe might need enhanced screening and targeted antibiotic protocols to address higher prevalence. Such tailored approaches could effectively reduce^14,18,19^ AR in these vulnerable populations.^28–30^ Moreover there is not a comprehensive research on the impact of AR on treatment outcomes in AD patients^14,18,19^ creating a knowledge gap which necessitates a systematic review and meta-analysis.^31,32^ To this end, our current review aims to investigate the prevalence of AR in older adults and AD patients with the goal to inform and provide some comprehensive healthcare approaches and interventions that are tailored to the specific needs of these groups.

## METHODS

A structured PRISMA reporting process was followed.^33^

### Eligibility criteria

The PEO framework is a structured approach to developing research questions and defining eligibility criteria for systematic reviews and meta-analyses.^34^ PEO stands for Population, Exposure, and Outcome. Table 1 outlines the eligibility criteria for this systematic review based on the PEO framework and provides a clear rationale for each criterion to help guide the study selection process.

**Table 1:** Eligibility criteria

Criteria	Inclusion criteria	Exclusion criteria
Population (P)	Older adults and AD patients	Patients without AD with other co-morbidities
Exposure (E)	Antibiotic resistance	Studies examining other health conditions or interventions
Outcome (O)	Prevalence of antibiotic resistance in the specified population	Studies not reporting prevalence of antibiotic resistance
Design	Observational studies (cross-sectional, cohort), randomized control trials, quasi-experimental studies, controlled designs	Qualitative studies, systematic review designs, narrative reviews, and commentaries
Publication Type	Published studies	Preprints
Language	English studies	Non-English

### Search strategy

The search strategy for this systematic review was developed using the PEO framework (Table 2). Key components include population (AD patients), exposure (antibiotic resistance), and outcome (prevalence of antibiotic resistance).^34^ Medical Subject Headings was used to supplement the primary keyword,^35,36^ and account for spelling variations. The primary keywords included “Alzheimer’s disease”, “older adults”, “antibiotic resistance,” and “prevalence”. Boolean operators such as AND, OR, and NOT were used to combine or exclude specific terms, thereby improving the search strategy.^37^ Wildcards, brackets, and truncation were used as additional search strings to prevalence of antibiotic resistance in AD patients ensuring a thorough and exhaustive review of the available evidence.^38^

**Table 2:** Search strategy

S /N	Primary keywords	Joining type	MESH Terms and Free Text with Boolean Operators	Databases
1	Patients with Alzheimer’s disease	OR	((“Patients with Alzheimer*”) OR (“Alzheimer Disease”) OR (“Alzheimer’s Disease”) OR (Alzheimer*) OR (Patient*) OR (“patient*, Alzheimer*”) OR (“Dementia, Alzheimer’s Type”) OR (“Early-Onset Alzheimer’s Disease”) OR (“Late-Onset Alzheimer’s Disease”) OR (“Senile Dementia”) OR (“Alzheimer’s, Presenile”)) OR (“older adults”) OR (“elderly patients”) OR (“elderly population”).	All Databases
		AND
2	Antibiotic resistance	OR	((“Antibiotic resistance”) OR (“Drug Resistance, Bacterial”) OR (“Antibiotics Resistance”) OR (“Drug Resistant Bacteria”) OR (“Antibiotics, bacteria, resistant”) OR (“Multidrug Resistance”) OR (“Antibiotic Resistant Infections”) OR (“Antimicrobial Resistance”) OR (“Resistance, Antibiotic”) OR (“Antimicrobial resistance”) OR (“Antimicrobial agent”) OR (“Antimicrobial drugs”) OR (“Antimicrobial therapy”) OR (“Antimicrobial treatment”) OR (“Antibacterial resistance”) OR (“Antibacterial agent”) OR (“Antibacterial drugs”) OR (“Antibacterial therapy”) OR (“Antibacterial treatment”) OR (“Tetracyclines”) OR (“Amphenicols”) OR (“Beta-lactam Antibacterials, Penicillins”) OR (“Other Beta-lactam Antibacterials”) OR (“Sulfonamides and Trimethoprim”) OR (“Macrolides, Lincosamides, and Streptogramins”) OR (“Aminoglycosides”) OR (“Quinolone Antibacterials”) OR (“Combinations of Antibacterials”) OR (“Other Antibacterials”))	All Databases
		AND
3	Prevalence	OR	((Prevalence*) OR (Prevalence) OR (rate) OR (“prevalent* rate”) OR (Incidence) OR (“Epidemiology”) OR (“Occurrence”) OR (“Frequency”) OR (“Distribution”))	All Databases

The databases used were PubMed, Scopus, CINAHL, and Web of Science.

### Study selection

The study selection process for this review began with the essential step of de-duplication, which was meticulously performed using the Zotero reference manager to ensure that each unique record was considered for inclusion.^39^ After de-duplication, a systematic screening process was employed.^40^ Initially, titles and abstracts were reviewed to identify potentially relevant studies based on predefined eligibility criteria, using the PEO framework. Revision was performed on the full text of the study articles and this was thoroughly done ensuring that they met the inclusion criteria and to extract relevant data.^40–42^

### Data extraction

To organize the data for this systematic review, references were transferred from Zotero to an Excel spreadsheet.^43–45^ The full-text articles were then used to extract comprehensive characteristics about the included studies. These included author names, publication year, study design, participant demographics, interventions or exposures related to antibiotic resistance, outcome measures (prevalence of antibiotic resistance), and relevant findings. Furthermore, information about the study’s duration, geographic location, sample size, and any statistical methods used for data analysis were systematically documented. The extraction also included information about the publication type, ensuring that only published studies were considered in the analysis.^46^

### Quality assessment

The quality assessment,^47,48^ also known as risk of bias evaluation,^49^ utilized the ROBINS-I tool (“Risk Of Bias In Non-randomized Studies - of Interventions”). The tool covers various important areas such as confounding variables, participant selection, intervention classification, deviations from intended interventions, missing data, outcome measurement, and selection of reported results. This was necessary as all the studies were non-randomized, and the ROBIN-I scale was considered the most suitable. This tool for quality assessment sought to provide a comprehensive evaluation of the internal validity of the studies, thereby contributing to the overall rigor of the systematic review.^49^

### Data synthesis

Based on the method of data synthesis, a meta-analysis approach was adopted random effect meta-analysis approach for quantitative synthesis was carried out for this review using Review Manager [RevMan] Software 5.4.1.^44,46,50–53^ The odds ratio and prevalence data were extracted from the studies. This method accounted for both within-study and between-study variability, yielding a more conservative estimate of the overall effect size.^54^ The I^2^ statistic was used to assess study heterogeneity, with values greater than 50% indicating significant heterogeneity.^55–57^ Also, Forest plots were generated to illustrate the results.

## RESULTS

**Fig. 1:**
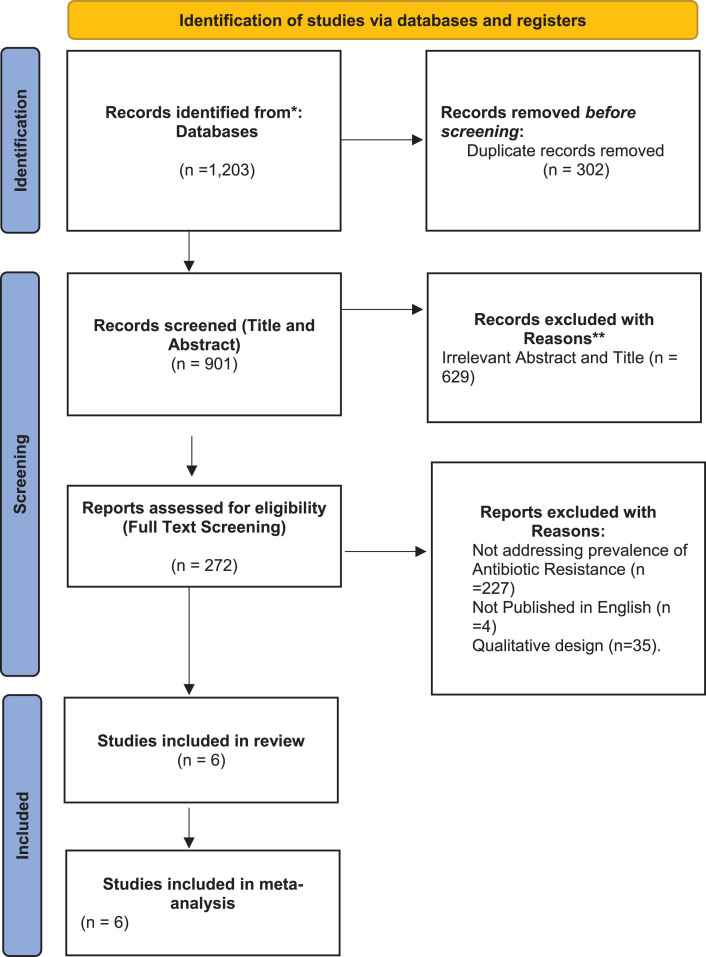
PRISMA flow diagram illustrating the study selection process.

A total of 1,203 studies were found following search from the databases, 272 studies were screened for full text assessment and at the end 6 studies met the full eligibility criteria for selection (Fig. 1).

### Characteristics of the included studies

A total of six studies were included in this review.^58–63^ Table 3 outlines the characteristics of the studies. The included studies, while diverse in their objectives and methods, share a focus on antibiotic resistance and its implications in various populations and settings. They encompass a range of study designs, from retrospective analyses (Weber et al., Washio et al.) to prospective evaluations (Eveillard et al., Fabiszewski et al.) and case-control studies (Wiener et al.). Sample sizes vary significantly, from small groups (Eveillard et al. with 35 patients) to large datasets (Weber et al. with 55,427 isolates). Key similarities include the identification of high antibiotic resistance prevalence, with MRSA and ESBL-producing bacteria being common focal points. Differences arise in participant characteristics, such as age groups (older versus younger adults, nursing home residents, AD patients), and in specific outcomes, like case fatality rates and treatment impacts. Limitations are consistently noted, including narrow population scopes and the challenge of establishing causation. Geographic and institutional contexts also vary, affecting the generalizability of findings. These studies collectively underscore the complexity of antibiotic resistance and the need for tailored interventions based on specific patient demographics and healthcare settings.

**Table 3:** Characteristics of the included studies

Study	Author publication date	Research objective	Research design &methods	Sample size or participant characteristics	Prevalence of antibiotic resistance	Outcome measures and findings	Location of study	Limitations
1	Weber et al. (2009)^62^	Compare antimicrobial resistance in older and younger adults	Retrospective analysis; Two-centre study	55,427 isolates between 1999–2005	11.82%	Heterogeneity observed; No uniform age-resistance association	Maryland and Chicago	Results vary by bacteria, age, and location; Temporal trends not specified
2	Eveillard et al. (2002)^61^	Estimate MRSA prevalence at admission	Prospective screening; Logistic regression analysis	35 patients; Demographics, clinical, and therapeutic data recorded	14.6% MRSA carriage; Associated with recent hospitalization and wounds	High MRSA prevalence at admission; Selective screening recommended	French teaching hospital	Limited to acute geriatric wards; Association, not causation
3	Washio et al. (1997)^59^	Evaluate factors influencing MRSA case fatality rate	Retrospective study; Univariate and multivariate analysis	49 elderly patients with MRSA infection	67.3% case fatality rate; Risk factors: male sex, hypoalbuminemia, excessive antibiotic usage	Case fatality rate may be high; Limited to a geriatric hospital	NR	NR
4	Wiener et al. (1999)^60^	Report nursing home outbreak of ESBL-producing gram-negative bacilli	Case-control study; Point-prevalence survey	55 patients; Clinical and molecular epidemiology	9.02%	Nursing home patients important reservoir; Common antibiotic resistance plasmid	Tertiary care hospital and community nursing home	Broad-spectrum antibiotics and poor infection control practices may facilitate spread
5	Fabiszewski et al. (1990)^58^	Impact of antibiotic treatment on fever outcomes in AD patients	Prospective evaluation; Survival analysis	104 institutionalized AD patients	11.02%	Antibiotic treatment does not alter fever outcome	NR	Limited to Alzheimer patients; Survival differences observed in less severely affected patients
6	Bello-Medina et al. (2022)^63^	Effect of BGM dysbiosis on AD patients	Preclinical study on 3xTg mice; Antibiotics induced BGM dysbiosis	102 patients	29.39%	Delay in spatial memory impairment and Aβ deposits; Correlated with bacterial abundance and alpha diversity	NR	Specific BGM effects may not be universally applicable

NR, not reported.

### Quality assessment

**Fig. 2:**
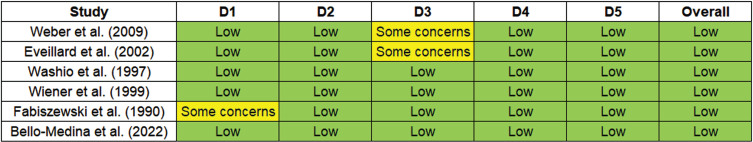
Risk of bias across the studies.

The ROBINS-I tool assesses the risk of bias in non-randomized studies of interventions. Each study is evaluated across different domains to determine the overall risk of bias. The several domains used were: 1) Bias due to confounding, which evaluates whether the effect of interest is mixed with other factors, 2) Bias in selection of participants, which examines whether the participants included in the study are representative, 3) Bias in classification of interventions, addressing potential misclassification of intervention status, 4) Bias due to deviations from intended interventions, assessing whether deviations from the planned interventions have occurred, 5) Bias due to missing data and in measurement of outcomes, evaluating whether the outcomes are measured appropriately. These domains collectively help in evaluating the overall risk of bias in non-randomized studies. For studies with Low: it implied the study has a low risk of bias across all domains. For moderate: the study has some concerns regarding bias, but the overall risk is moderate. And for High: the study has a high risk of bias, indicating significant concerns across multiple domains. Figure 2 illustrates the Risk of bias across studies.

### Meta-analysis of prevalence of AR in older adults and AD

**Fig. 3:**
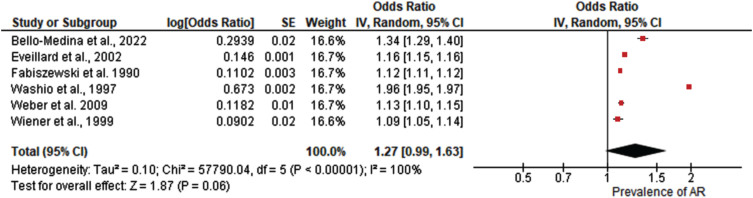
Forest plot demonstrating the pooled prevalence of antibiotic resistance in older adults and Alzheimer’s disease patients.

A total of six studies were included in the meta-analysis of pooled prevalence of antibiotic resistance in older adults and AD patients.^58–63^ Results from the study revealed as shown in Fig. 3 that the pooled prevalence was (OR = 1.27; 95% C.I: [0.99, 1.63] *Z* = 1.87, *p* = 0.06). Significant heterogeneity was observed among the studies (I^2^ = 100%, *p* < 0.00001).

## DISCUSSION

The objective of this systematic review and meta-analysis was to ascertain the prevalence of antibiotic resistance in older adults and AD patients. A meta-analysis of six selected studies found a pooled prevalence odds ratio (OR) of 1.27 (95% CI: [0.99, 1.63], *Z* = 1.87, *P* = 0.06), indicating a possible trend toward increased antibiotic resistance, though statistical significance was not reached. This analysis revealed significant heterogeneity among the studies (I^2^=100%, *p* < 0.00001), indicating substantial variation in methodologies and participant characteristics. This high level of heterogeneity necessitates careful interpretation of the results, as the differences between studies can influence the overall findings. Heterogeneity in a meta-analysis often arises from variations in study design, population demographics, intervention types, and outcome measurements.^14^ Such diversity can obscure true effects and complicate the synthesis of findings. For instance, studies may differ in their definitions of antibiotic resistance, the diagnostic criteria for AD, or the healthcaresettings in which data were collected.^6,19,22^

Discussing the effects of this heterogeneity is crucial, as it impacts the robustness and applicability of the meta-analysis results. Differences and similarities between studies were explored to understand the sources of variation and their implications and this variations in resistance trends across bacterial species, geographical locations, and healthcare settings highlight the need for targeted, context-specific interventions were seen. Comparing these findings with existing literature reveals key trends in antibiotic resistance, but the relevance to the current study’s results was noted.^12^ The variability underscores the necessity for nuanced strategies in combating antibiotic resistance, tailored to specific contexts. The authors however emphasize how their results align with or diverge from established literature, reinforcing the importance of customized interventions in addressing the global challenge of antibiotic resistance.^33,39^ This would provide a unifying theme and demonstrate the significance of their meta-analysis in the broader context of existing research. Paczosa and Mecsas discuss Klebsiella pneumoniae’s defensive mechanisms, emphasizing the changing nature of bacterial resistance strategies.^64^ Reyes et al. offer microbiological insights into carbapenem-resistant K. pneumoniae, emphasizing the clinical significance of antibiotic resistance.^65^ Zhang et al. emphasize the urgent need for antimicrobial stewardship in tertiary hospitals, which is consistent with the broader concerns raised in the systematic review.^66^ Li et al. describes a rapid increase in the prevalence of carbapenem-resistant Enterobacteriaceae (CRE) and the emergence of colistin resistance in a Chinese hospital, highlighting a global concern echoed in the meta-analysis.^67^ Park et al. present a downward trend in carbapenem-resistant K. pneumoniae infections in New York City hospitals, which contrasts with the potential trend suggested in the current review.^68^ Also, Morgenstern et al. investigate antibiotic resistance in commensal Staphylococcus aureus, providing a more comprehensive understanding of resistance patterns beyond Enterobacteriaceae.^69^ Kajumbula et al. in Lacor Hospital, Uganda, and Chen et al. in a regional burn center in China investigated antibiotic susceptibility in various healthcare settings.^70,71^ Also, Brooks and Mias investigate Streptococcus pneumoniae virulence and host immunity, addressing the larger context of bacterial infections and antibiotic resistance.^72^ However, while the systematic review provides useful insights into antibiotic resistance in older adults and AD patients, a critical comparison to existing literature highlights the complex and multifaceted nature of antibiotic resistance patterns. The variability in resistance trends across bacterial species, geographical locations, and healthcare settings emphasizes the need for targeted and context-specific interventions in the global fight against antibiotic resistance. Our current review, with its acknowledgement of significant heterogeneity, calls for additional research to identify the specific factors influencing resistance prevalence in these vulnerable populations.

This meta-analysis, which uncovered a potential trend of increased antibiotic resistance in older adults and AD patients, has important implications for clinical and public health practices. The observed heterogeneity among studies emphasizes the importance of tailoring interventions to the various contexts that influence resistance patterns. These findings highlight the importance of judicious antibiotic use in clinical settings, emphasizing the need for individualized treatment regimens and regular resistance pattern surveillance. Antibiotic resistance is a multifaceted issue that requires public health policies to integrate infection prevention, antimicrobial stewardship, and surveillance strategies. Because vulnerable populations, such as older adults with comorbidities, are especially vulnerable to the effects of antibiotic-resistant infections, these findings call for comprehensive public health measures to prevent the emergence and spread of resistant strains. Continuous monitoring, education, and collaboration among healthcare providers, researchers, and policymakers are critical in developing effective strategies to combat antibiotic resistance and protect the health of these communities.

Despite contributing valuable insights, our meta-analysis has certain limitations. Firstly, the availability of a limited number of studies on the topic restricted our ability to conduct a more extensive and robust analysis. The scarcity of data poses a challenge in establishing a comprehensive understanding of the antibiotic resistance landscape in older adults and AD patients. Furthermore, the substantial heterogeneity observed across the included studies raises concerns about the consistency of the findings. Variations in methodologies, participant demographics, and regional factors may have introduced confounding variables, affecting the reliability of the overall pooled estimate. Additionally, the lack of standardized reporting across studies may have impacted the accuracy and comparability of the results. As such, these limitations underscore the need for more well-designed and standardized research in this field to enhance the reliability and generalizability of future meta-analyses on antibiotic resistance in the context of older adults and AD patients.

### Conclusions

In conclusion, our meta-analysis of the prevalence of antibiotic resistance in older adults and AD patients revealed a complex landscape with a suggestive but statistically insignificant trend toward increased resistance. The significant heterogeneity among the included studies highlights the importance of exercising caution when interpreting the overall pooled estimate, taking into account different methodologies and participant characteristics. The implications of these findings highlight the complexities of antibiotic resistance among vulnerable populations. Clinical practices should prioritize individualized treatment strategies and antimicrobial stewardship, while public health policies should implement comprehensive measures to address the multifaceted nature of resistance. Further research is needed to identify the specific factors influencing resistance patterns in these populations, allowing for the development of targeted interventions. This study adds to the ongoing discussion about antibiotic resistance by emphasizing the importance of collaborative efforts in clinical and public health settings to reduce the emergence and spread of antibiotic-resistant strains among older adults and ADpatients.

## AUTHOR CONTRIBUTIONS

Namra Vinay Gohil (Conceptualization; Data curation; Writing – original draft; Writing – review & editing); Fabio Fuentes Gandara (Conceptualization; Data curation; Writing – original draft; Writing – review & editing); Harshal Gohil (Conceptualization; Data curation; Writing – original draft; Writing – review & editing); Swathi Gurajala (Conceptualization; Data curation; Writing – original draft; Writing – review & editing); David Chinaecherem Innocent (Conceptualization; Data curation; Writing – original draft; Writing – review & editing); Tadele Tesfaye (Conceptualization; Data curation; Writing – original draft; Writing – review & editing); Domenico Pratico (Conceptualization; Writing – review & editing).

## Data Availability

Data sharing is not applicable to this article as no datasets were generated or analyzed during this study.
